# Numerical Modelling of the Effect of Filler/Matrix Interfacial Strength on the Fracture of Cementitious Composites

**DOI:** 10.3390/ma11081362

**Published:** 2018-08-06

**Authors:** Xiaowei Ouyang, Zichao Pan, Zhiwei Qian, Yuwei Ma, Guang Ye, Klaas van Breugel

**Affiliations:** 1 Guangzhou University—Tamkang University Joint Research Center for Engineering Structure Disaster Prevention and Control, Guangzhou University, Guangzhou 510006, China; xwouyang@gzhu.edu.cn; 2 Faculty of Civil Engineering and Geosciences, Delft University of Technology, 2628CN Delft, The Netherlands; G.Ye@tudelft.nl (G.Y.); K.vanBreugel@tudelft.nl (K.v.B.); 3 Department of Bridge Engineering, Tongji University, Shanghai 200092, China; z.pan@tongji.edu.cn

**Keywords:** filler, interface, mechanical properties, blended cement paste, lattice fracture model

## Abstract

The interface between filler and hydration products can have a significant effect on the mechanical properties of the cement paste system. With different adhesion properties between filler and hydration products, the effect of microstructural features (size, shape, surface roughness), particle distribution and area fraction of filler on the fracture behavior of a blended cement paste system is supposed to be different, as well. In order to understand the effect of the microstructural features, particle distribution and area fraction of filler on the fracture behavior of a blended cement paste system with either strong or weak filler-matrix interface, microscale simulations with a lattice model are carried out. The results show that the strength of the filler-matrix interface plays a more important role than the microstructural features, particle distribution and area fraction of filler in the crack propagation and the strength of blended cement paste. The knowledge acquired here provides a clue, or direction, for improving the performance of existing fillers. To improve the performance of fillers in cement paste in terms of strength, priority should be given to improving the bond strength between filler particles and matrix, not to modifying the microstructural features (i.e., shape and surface roughness) of the filler.

## 1. Introduction

Portland cement is a basic component of concrete with a high environmental impact because of high CO_2_ emissions and energy consumption during production. Fillers such as limestone or quartz powder are used as a replacement for Portland cement to make concrete less expensive and more environment friendly [1,2,3]. Additions of limestone or quartz powder have a small chemical effect on cement hydration [4,5]. Finer fillers in cement paste can result in improvements in strength because of a denser packing [6]. However, the use of fillers results in dilution of Portland cement particles in the paste. Above a critical amount of fillers, this ‘dilution’ effect will lead to a lower strength of the hardened paste or concrete [1,7,8]. Besides, the mechanical properties of the interface between filler and hydration products have a significant influence on the development of the fracture process in blended cement paste under a compressive/tension load and, thus, the strength of the cement paste blended with fillers [9,10].

In order to predict the effect of the micromechanical properties of the interface on the fracture process in the cement paste blended with fillers, a numerical model was applied. This model, called the lattice fracture model, was proposed by Schangen and Van Mier in the 1990s and has been further developed by many others [11,12,13]. In the lattice fracture model, the material is represented by a lattice of beam elements. Subsequently, the microstructure of the material can be mapped onto these beam elements by assigning them different properties, depending on whether the beam element represents a grain, interface or matrix. Various conventional laboratory experiments can be simulated by the lattice fracture model, and the model can be applied to cement-based materials [14,15,16]. This model was also used to study the interface fracture in cement-based materials [17,18]. In previous studies [19], the lattice fracture model was used to study the influence of interface properties on crack propagation, tensile strength and fracture energy. The output from the simulated direct tension test was compared with experimental results and used to predict the micromechanical properties of the interface.

In previous studies [19,20,21], it was found that the strength of the interface between quartz particles and hydration products was so low that it has no noticeable contribution to the strength of the paste. By contrast, the strength of the interface between limestone particles and hydration products was suggested to be equal to or higher than that of the matrix itself. This is because the limestone has better adhesion properties than quartz. Besides adhesion properties, the microstructural features (size, shape, surface roughness), particle distribution and area fraction of filler may also play an important role in the strength and the fracture behavior of blended cement paste. These roles are not easy to measure experimentally. With the lattice fracture model, one can effectively simulate the micro-mechanical behavior of blended cement paste in order to gain insight into the effect of microstructural features, particle distribution and area fraction of filler on the strength and the fracture behavior of blended cement paste. The results of these simulations will enable us to get a better understanding of the role of filler-hydrates adhesion properties and their influence on the strength of blended cement paste. In this paper, microscale simulations with the lattice model are presented. The effect of microstructural features, particle distribution and volume fraction of filler on the fracture behavior of blended cement paste with weak and strong filler-matrix interfaces will be studied. The strong filler-matrix interface simulates the interface between limestone particles and hydration products, whereas the weak interface simulates the interface between quartz particles and hydration products.

## 2. Approach

### 2.1. Generation of Microstrocture

The simulated cement paste system refers to a hardened cement paste, in which the porosity is low and most of the clinker grains have been hydrated. Therefore, in the lattice approach, hardened cement paste with filler is considered a material made only of two components. Besides the matrix phase, only the fillers are represented explicitly. The unhydrated cement, hydration products and other components are assumed to be part of the matrix phase.

The circular particle is used as referential filler particle. It is assumed to have an identical radius r. Then, the total number of fillers can be simply calculated according to the model size, area fraction of the filler and the identical filler radius,

(1) $$N=\langle \frac{H^{2}\Phi }{\pi r^{2}}\rangle$$

where *N* is the number of filler particles, *H* is the model size and *Φ* is the area fraction of the filler.

As solid particles, fillers are not allowed to be overlapped with one another. Thus, a packing algorithm is needed to determine the proper positions of fillers in the cement paste. In this paper, we used the classical “take-and-place” algorithm proposed by Wang et al. [22]. For each filler, a potential position is randomly generated. Then, the position is checked to see whether the filler is overlapped with any other fillers that have already been placed in the cement paste. If not, the potential position is accepted, and the next filler is placed. Otherwise, another potential position is tried until the proper position of the filler is found. If the filler is modeled as a polygon, a reference circle with an identical radius is firstly formed. Then, a polygon is randomly generated by cutting, stretching and scaling the reference circle. The periodic boundary condition is used in the particle placement algorithm. A detailed description of this procedure can be found in Pan et al. [23,24].

### 2.2. Construction of Lattice Structure

**Figure 1:**
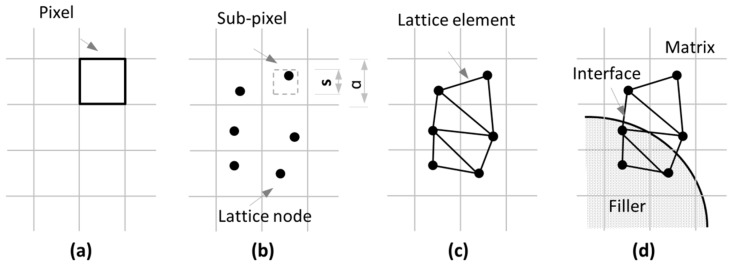
Schematics of microstructure generation [19]. (**a**) Pixel generation; (**b**) node placement; (**c**) element generation; (**d**) overlay procedure.

For the mesh generation, the microstructure of blended cement paste is represented by a pixel-based structure, as shown in Figure 1a. After the generation of a network of pixels, a sub-pixel can be defined with each pixel. A node is randomly placed in every sub-pixel of size s in a regular grid with size a, as shown in Figure 1b. The ratio s/a is defined as the randomness of a lattice. The reason for assigning certain randomness to the lattice mesh is to get rid of artificial periodic lattice effects and to describe the material more correctly. Here, randomness is set to be 0.5. To connect the nodes, lattice elements are generated using Delaunay triangulation, as shown in Figure 1c. The different types of beams are identified by material overlap procedures. Element properties depend on the two nodes assigned to an element. If the two element nodes are located within filler, the filler element can be identified. If one element node is located in the matrix and another element node located in hydration products, the element is called an interface element. In Figure 1d, the identification of different elements is presented. In this model, we generated a square specimen with an external size of 100 × 100 µm^2^ for a 2D lattice model.

### 2.3. Fracture Process Simulation

#### 2.3.1. Numerical Approach

**Figure 2:**
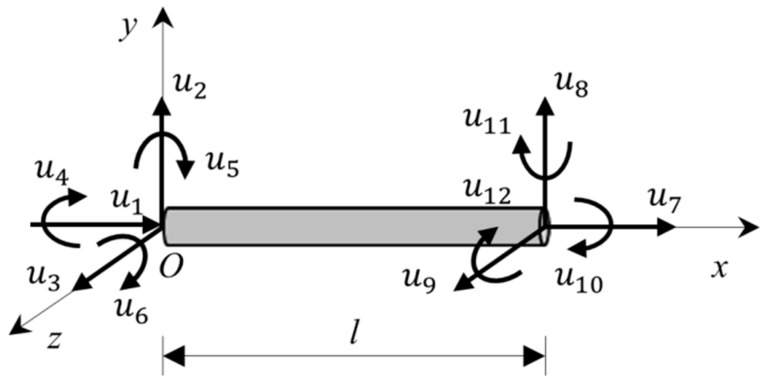
The displacement of lattice beam elements.

In the lattice approach, the cement paste blended with fillers is discretized as a set of lattice beam elements. A lattice beam element is a straight bar of uniform cross-section and can transmit axial forces, shear forces, bending moments and torsional moments. The lattice beam element has two nodes, and each node has six degrees of freedom including three translational and three rotational degrees of freedom, as shown in Figure 2. The displacement *u^e^* of the nodes of the beam in the local domain is:

(2) $$u^{e}={\left[u_{1}\;u_{2}\;u_{3}\;u_{4}\;u_{5}\;u_{6}\;u_{7}\;u_{8}\;u_{9}\;u_{10}\;u_{11}\;u_{12}\right]}^{T}$$

The local force vector *f^e^* caused by local displacement *u^e^* can be computed by the following equation:

(3) $$f^{e}=k^{e}u^{e}$$

where *k^e^* is the element stiffness matrix in the local domain. The formulation of a lattice beam element stiffness matrix is based on the Timoshenko beam theory. It can be formulated by assembling the axial component, torsional component, bending and shear components in the planes xOy and xOz and given by:

(4) $$k^{e}=[\begin{array}{cccccccccccc} \frac{EA}{l} & 0 & 0 & 0 & 0 & 0 & -\frac{EA}{l} & 0 & 0 & 0 & 0 & 0\\ & \frac{12EI_{z}}{l^{3}\left(1+\Phi_{1}\right)} & 0 & 0 & 0 & \frac{6EI_{z}}{l^{2}\left(1+\Phi_{1}\right)} & 0 & -\frac{12EI_{z}}{l^{3}\left(1+\Phi_{1}\right)} & 0 & 0 & 0 & \frac{6EI_{z}}{l^{2}\left(1+\Phi_{1}\right)}\\ & & \frac{12EI_{y}}{l^{3}\left(1+\Phi_{1}\right)} & 0 & -\frac{6EI_{y}}{l^{2}\left(1+\Phi_{2}\right)} & 0 & 0 & 0 & -\frac{12EI_{y}}{l^{3}\left(1+\Phi_{2}\right)} & 0 & -\frac{6EI_{y}}{l^{2}\left(1+\Phi_{2}\right)} & 0\\ & & & \frac{GJ}{l} & 0 & 0 & 0 & 0 & 0 & -\frac{GJ}{l} & 0 & 0\\ & & & & \frac{\left(4+\Phi_{2}\right)EI_{y}}{l\left(1+\Phi_{2}\right)} & 0 & 0 & 0 & \frac{6EI_{y}}{l^{2}\left(1+\Phi_{2}\right)} & 0 & \frac{\left(2-\Phi_{2}\right)EI_{y}}{l\left(1+\Phi_{2}\right)} & 0\\ & & & & & \frac{\left(4+\Phi_{1}\right)EI_{z}}{l\left(1+\Phi_{1}\right)} & 0 & -\frac{6EI_{z}}{l^{2}\left(1+\Phi_{1}\right)} & 0 & 0 & 0 & \frac{\left(2-\Phi_{2}\right)EI_{z}}{l\left(1+\Phi_{1}\right)}\\ & & & & & & \frac{EA}{l} & 0 & 0 & 0 & 0 & 0\\ & & & & & & & \frac{12EI_{z}}{l^{3}\left(1+\Phi_{2}\right)} & 0 & 0 & 0 & -\frac{6EI_{z}}{l^{2}\left(1+\Phi_{1}\right)}\\ & & & & & & & & \frac{12EI_{y}}{l^{3}\left(1+\Phi_{2}\right)} & 0 & \frac{6EI_{y}}{l^{2}\left(1+\Phi_{2}\right)} & 0\\ & & & & & & & & & \frac{GJ}{l} & 0 & 0\\ & & & & & & & & & & \frac{\left(4+\Phi_{2}\right)EI_{y}}{l\left(1+\Phi_{2}\right)} & 0\\ & & & & & & & & & & & \frac{\left(4+\Phi_{1}\right)EI_{z}}{l\left(1+\Phi_{1}\right)} \end{array}]$$

where *E* is the elastic modulus, *G* is the shear modulus, *A* is the cross-sectional area of the element, l is the length of the element, *I_z_* and *I_y_* are the moment of inertia about the *z*-axis and the *y*-axis, respectively, *J* is the polar moment of inertia about the *x*-axis and *Φ*_1_ and *Φ*_2_ are the shear effect adjustment factors in the plane xOy and xOz, respectively, and can computed by:

(5) $$\;\{\begin{array}{c} \Phi_{1}=\frac{12{El}_{z}}{{GA}_{s}l^{2}}\\ \Phi_{2}=\frac{12{El}_{y}}{{GA}_{s}l^{2}} \end{array}\;$$

in which *A_s_* is the shear cross-sectional area and is given by:

(6) $$\;A_{s}=\;\frac{A}{k}\;$$

where *k* is the shear correction factor and is equal to $\frac{10}{9}$.

In lattice network construction, a circular cross-section of the element is preferred to a rectangular one, as it gives isotropic geometry properties about the cross-section, and the following formulas apply,

(7) $$\;\{\begin{array}{c} \begin{array}{c} A={\pi r}^{2}\\ I_{z}=\frac{\pi }{4}r^{4} \end{array}\\ \begin{array}{c} I_{y}=\frac{\pi }{4}r^{4}\\ J=\frac{\pi }{2}r^{4} \end{array} \end{array}\;$$

#### 2.3.2. Geometry and Boundary Conditions

**Figure 3:**
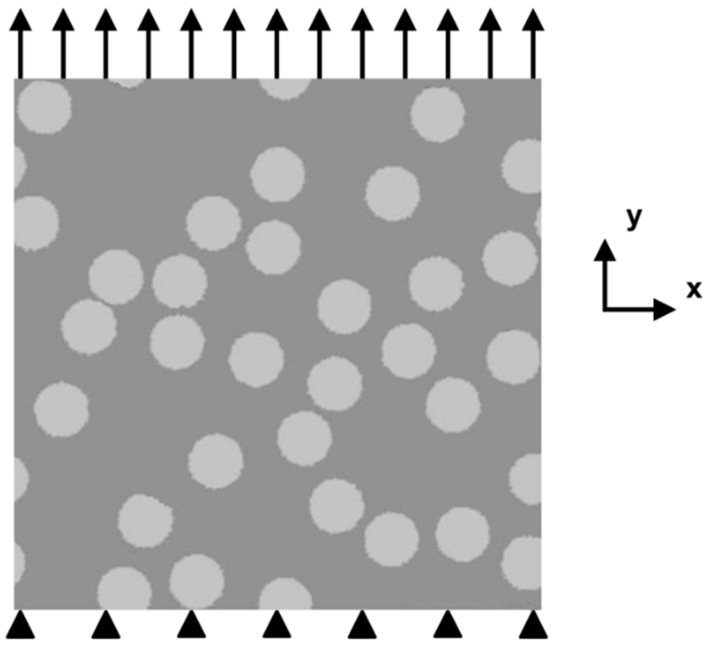
Uniaxial tensile test setup.

A uniaxial tensile test can be set up by fixing all the nodes on the bottom line of the specimen and imposing a uniform surface load on the top line, as shown in Figure 3. During the lattice network construction, all the layers close to the surfaces are forced to be regularly meshed, irrespective of the material randomness setting, as irregular geometry on the boundaries might create some extra stresses, which may have a negative effect on the fracture process simulation. In addition, all the lattice elements involved in the bottom and top layers are not allowed to be broken, even if the stress in such an element has already exceeded its strength, as the external loads need a path to be transferred into the specimen. In other words, it is assumed that all the restraint elements have infinite strength.

#### 2.3.3. Fracture Law

In lattice fracture analysis, a linear elastic analysis of the lattice is performed in each loading step. An element in which a prescribed fracture criterion is exceeded is removed from the mesh. This analysis is subsequently repeated in a step-wise manner, removing a single element in each step. Multiple analysis steps are performed until the system fails. The step-by-step removal of critical lattice elements simulates the microcrack evolution in the specimen. In this model, a fracture criterion based on the tensile stress in beams is adopted and can be described as:

(8) $$\sigma t\;=\alpha_{N}\frac{N}{A}\;+\alpha_{M}\frac{\max\;(M_{i},{\;M}_{j})}{w}\;\leq \;f_{t,k}$$

where *σ*_t_ (kPa) is the maximum tensile stress in the beam, *N* (kN) is the normal force acting on the beam element, *M_i_* (kN·m) and *M_j_* (kN·m) are the bending moments in two nodes at each end of the element and *w* (m^3^) is the cross-sectional moment of resistance. $\alpha_{N}$ and $\alpha_{M}$ are the normal force influence factor and the bending influence factor, the values of which are 1.0 and 0.05, respectively.

### 2.4. Mesh Sensitivity

Different mesh sizes may cause variations in the simulation results such as the crack pattern. Therefore, the mesh size should be chosen with care. In previous studies [19], the influence of the mesh size had been studied. A series of numerical tests has been carried out with different mesh sizes. The results have shown that the pixel size under 0.5 µm did not have a significant influence on the crack pattern. In order to save some computational time, the cell size for the simulations presented in this study was set to 0.4 µm.

### 2.5. Material Parameters

A lattice beam is a discretization of a continuum. The number of forces that can be transmitted by the elements of a lattice determines the type of continuum that is represented by the lattice. The lattice beams need to be fed with specific material parameters. In particular, fracture properties of the three types of beam elements are needed, including properties of the filler, matrix and filler-matrix interface. In real cement paste, the value of those parameters has a significant influence on the microscopic mechanical behavior. In particular, the strength of the filler-matrix interface directly affects the final results, i.e., crack patterns, stress-strain curve and Young′s modulus [25,26]. The elastic properties of fillers and matrix are inferred from the results of nanoindentation tests. In Table 1, a summary of the mechanical parameters of chosen fillers (quartz and limestone) and the matrix is given. The data for mechanical properties of interface elements are from [19].

## 3. Results and Discussion

In order to study the effect of filler/matrix interfacial strength on the plastic-damage of cementitious composites, the effect of particle distribution, size, shape, surface roughness and area fraction of filler on the fracture of blended cement paste with different adhesion properties between filler and hydration products is investigated. The particle distribution, size, shape, surface roughness and area fraction of filler in referential specimen are randomly distributed, 10 µm, circular, smooth and 25%, respectively.

### 3.1. Effect of Filler Distribution

**Figure 4:**
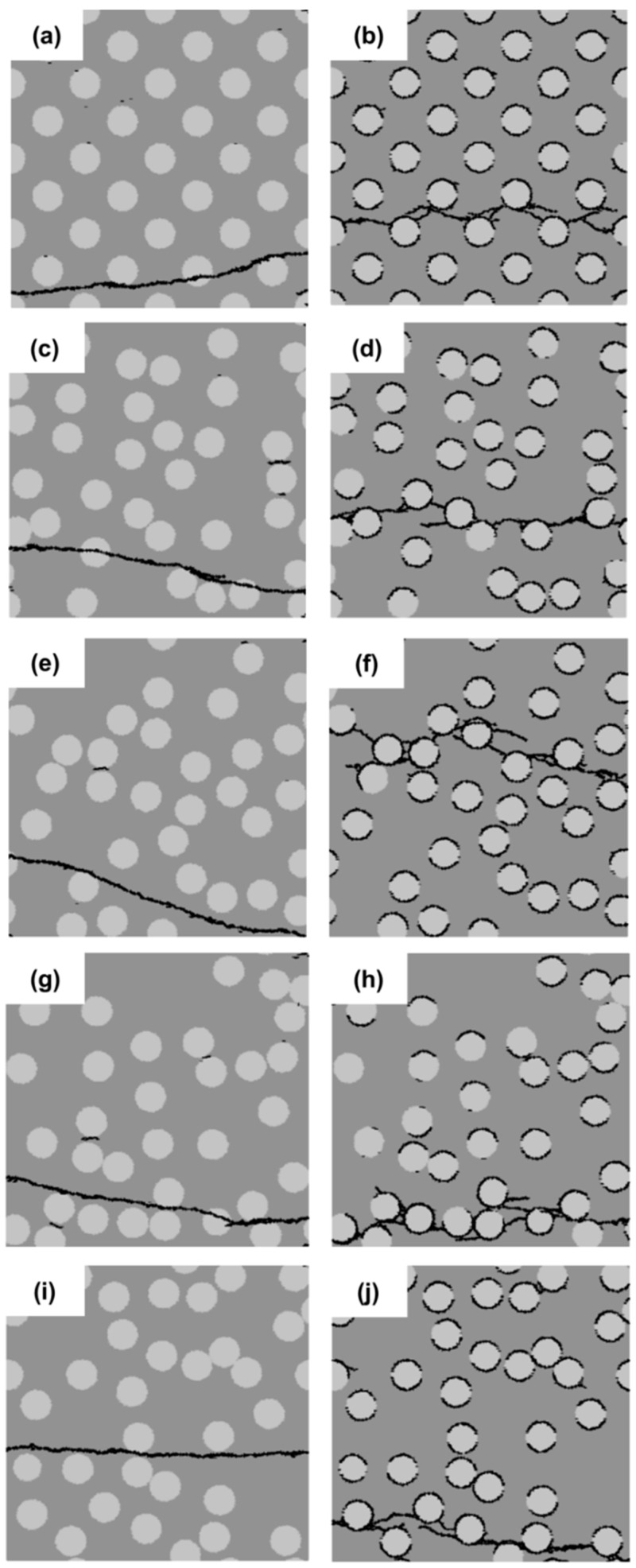
Micro-crack patterns of specimens with a strong interface (left) and a weak interface (right) for five different filler particle distributions (Distribution 1 (**a**,**b**), Distribution 2 (**c**,**d**), Distribution 3 (**e**,**f**), Distribution 4 (**g**,**h**) and Distribution 5 (**i**,**j**)).

The concentration of the filler particles in a localized region may have an effect on the strength of cement paste. In this section, five different distributions of filler particles are considered and divided into two groups, as shown in Figure 4. The left column in Figure 4 represents the group with a strong interface (SI); the right column is that with a weak interface (WI). The filler particles in the specimen of Figure 4a,b are uniformly distributed. The filler particles in the rest of the specimens are randomly distributed. The diameter of filler particles is 10 µm. The filler area fraction is 25%.

**Figure 5:**
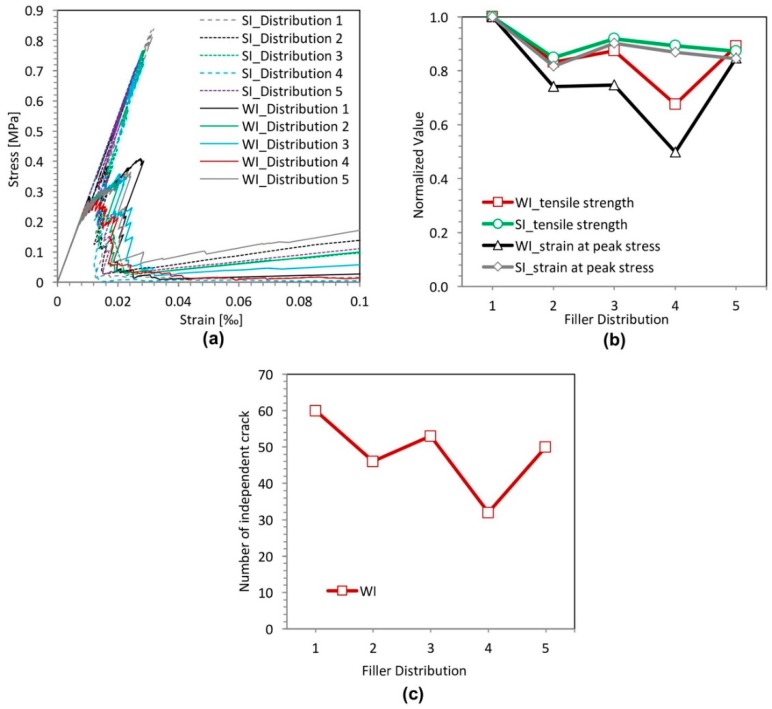
Simulated results of the filler distribution effect analyses for specimens with a strong interface (SI) and a weak interface (WI). (**a**) Stress-strain relation; (**b**) variation of the strength and the strain at peak stress; (**c**) number of independent cracks.

The micro-crack patterns for the different cement paste specimens with strong interface (SI) and weak interface (WI) are shown in Figure 4. Those figures show that the crack patterns in the left group and that in the right are very different. In the left group with the strong interface, there is only one main crack, and the crack pattern does not change, regardless of the distribution of filler particles; whereas, a large number of branched and tortuous cracks can be seen in the specimens with the weak interface (WI). With different particle distributions, the crack pattern in the specimens with the weak interface (WI) has a change, especially in the number of independent cracks, as shown in Figure 5c. The specimen with uniformly-distributed filler particles has the highest number of independent crack. This is because stresses are uniformly distributed across the cross-section of these specimens with uniformly-distributed filler particles. Besides, from Figure 4a,c,e,g, it can be observed that occasionally, the crack propagates through the filler particle instead of propagating along the filler particle. This is due to the strong interface and a favorable direction for the crack propagating into the filler particle.

Figure 5a,b plots the stress-strain relations and variation of the strength and the strain at peak stress for different filler particle distributions with a strong interface (SI) and a weak interface (WI). From these graphs, it can be inferred that the strength of specimens with a weak interface (WI) is always much lower compared to that of specimens with a strong interface (SI). The specimens with the uniformly-distributed filler particles have the highest strength because stresses are uniformly distributed across the cross-section of these specimens. However, in practice, it is impossible to achieve such a uniform distribution. For the specimens with randomly-distributed filler particles, the influence of the filler distribution on the strength of the specimens with a strong interface (SI) is much smaller compared with that with the weak interface (WI). With different particle distributions, the specimens with a weak interface (WI) have a very different strength. The strength correlates with the number of independent cracks, as can be observed in Figure 5b,c. The specimen with a weak interface (WI) and filler particle Distribution 4 has the lowest strength and the least independent cracks, as shown in Figure 5b,c. This is due to the fact that many filler particles in this specimen are concentrated in a small region and very close to each other in the horizontal direction, as shown Figure 4h. This facilitates the connection of cracks and the final damage leading to lower strength; whereas, in the specimen (Figure 4b), all the initial cracks are spread out and developed because the filler particles are uniformly distributed. In this case, a higher stress is needed to reach the destruction point. For the specimen with a strong interface (SI), the initiation of cracks can be stopped due to high local cohesive strength. Therefore, only one main crack is developed.

### 3.2. Effect of Filler Size

**Figure 6:**
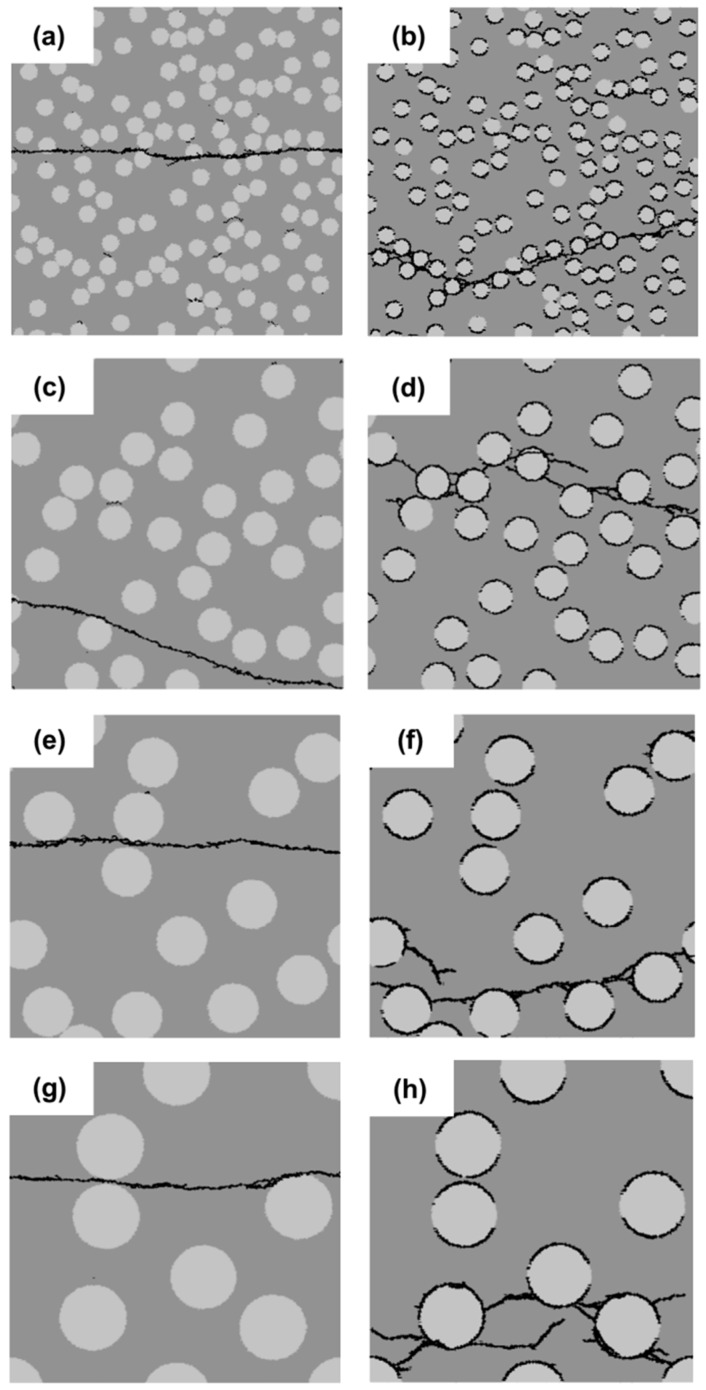
Micro-crack patterns of specimens with randomly-distributed filler particles for four different filler particle sizes (5 µm (**a**,**b**), 10 µm (**c**,**d**), 15 µm (**e**,**f**) and 20 µm (**g**,**h**)).

**Figure 7:**
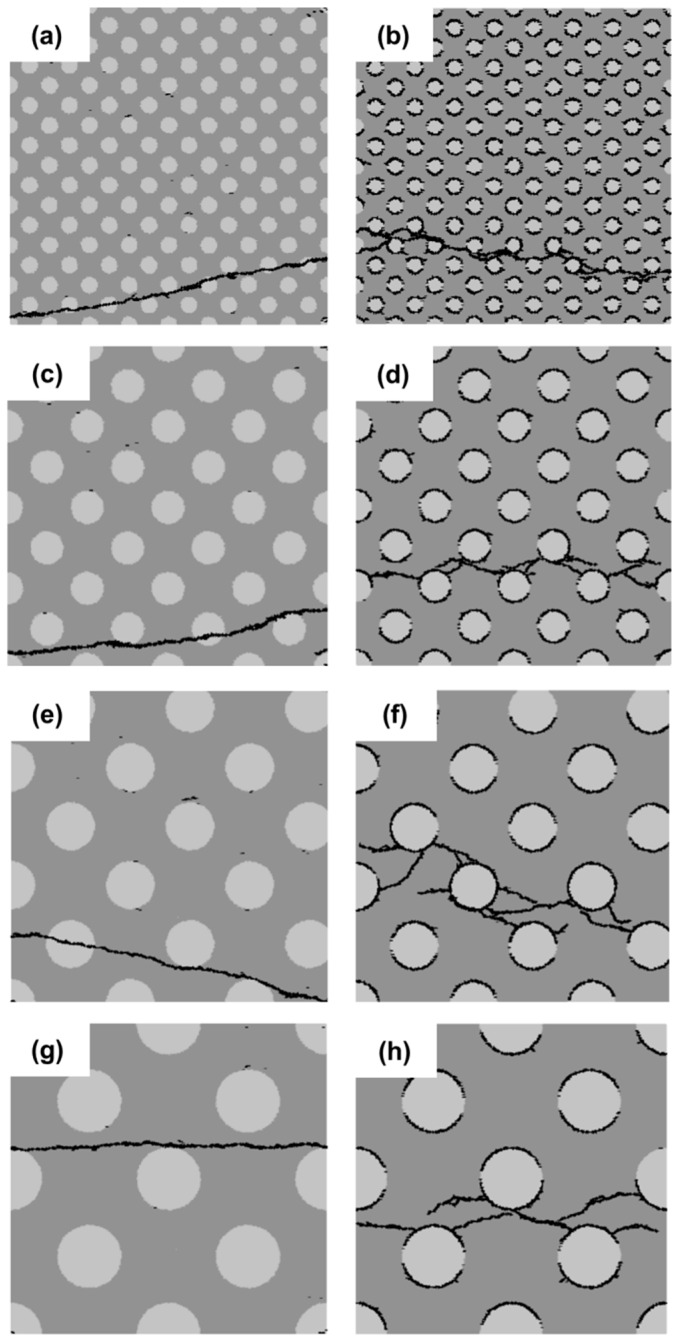
Micro-crack patterns of specimens with uniformly-distributed filler particles for different filler particle sizes (5 µm (**a**,**b**), 10 µm (**c**,**d**), 15 µm (**e**,**f**) and 20 µm (**g**,**h**)).

In this section, the effect of the filler particle size on the strength and the fracture behavior of blended cement paste is analyzed. Four different particle sizes (5 µm, 10 µm, 15 µm and 20 µm) are considered. They are divided into two groups as indicated in Figure 6 and Figure 7: on the left is the group with a strong interface (SI); on the right is that with a weak interface (WI). The filler area fraction is kept constant at 25%. With increasing particle size, the number of filler particles decreases. The filler particles in Figure 6 are randomly distributed. As a reference, the filler particles presented in Figure 7 are uniformly distributed.

Figure 6 and Figure 7 show the micro-crack patterns for the different cement paste specimens with a strong filler-matrix interface (SI) and a weak filler-matrix interface (WI). Within the same group (left or right group), there is a fine distinction between the micro-crack patterns of different particle distributions. However, there is a big difference between the micro-crack patterns of the left and right group. From these graphs, it can be inferred that the distribution of filler particles has a smaller effect on the crack patterns compared to the effect of interfacial strength.

**Figure 8:**
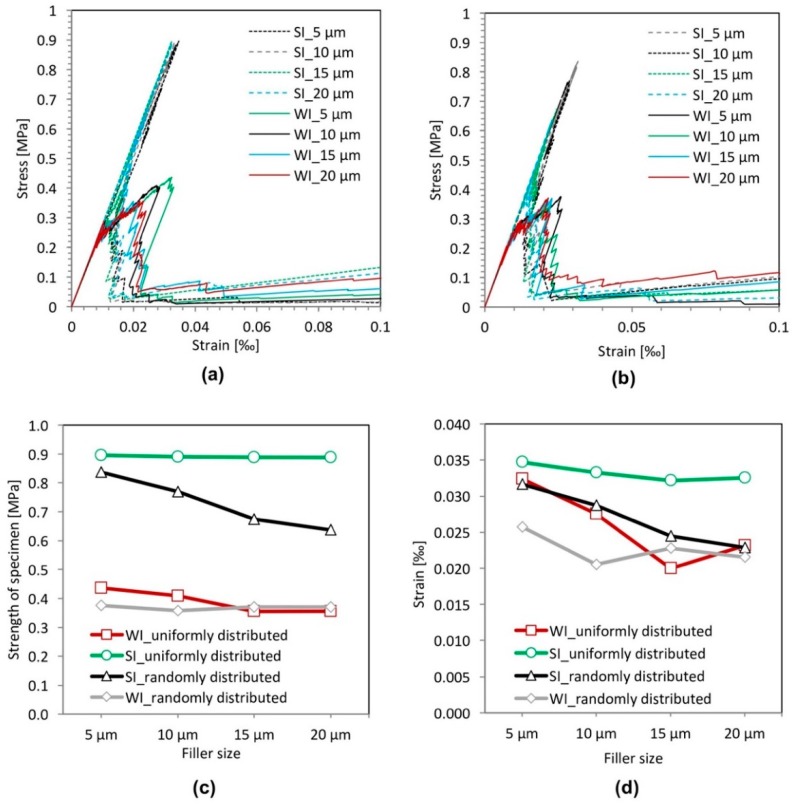
Simulated results of the filler size effect analyses for specimens with a strong interface (SI) and a weak interface (WI). (**a**) Stress-strain relation (uniformly distributed); (**b**) stress-strain relation (randomly distributed); (**c**) strength; (**d**) strain at peak stress.

Figure 8 plots the stress-strain relations, the strength and the strain at peak stress for different filler particle sizes with a strong filler-matrix interface (SI) and a weak filler-matrix interface (WI). Figure 8c shows that for the cement paste specimens with a strong filler-matrix interface (SI), the influence of the particle size on the strength of the specimen is insignificant in the case of a uniform distribution of filler particles, whereas in the case of a random distribution of filler particle, the strength of the specimen decreases with increasing particle size. This may be caused by the increasing stress concentration due to increasing filler size when the filler particles are randomly distributed. This stress concentration facilitates the initiation of cracks and the further propagation of cracks, leading to lower strength; whereas, in the case of a uniform distribution of filler particles, stresses are uniformly distributed across the cross-section of the specimens.

For the specimens with a weak filler-matrix interface (WI), the effect of particle size on the strength of specimen is not apparent regardless of the spatial distribution of filler particles if compared to the effect of particle size on the strength of specimens with a strong interface. Looking closely (Figure 8c), the influence of the particle size on the strength of the specimen is insignificant in the case of a random distribution of filler particles, whereas in the case of uniform distribution of filler particles, the strength of the specimen decreases slightly with increasing particle size. This is contrary to the situation in the case of the cement paste specimens with a strong filler-matrix interface (SI). This may due to the fact that the specimen with smaller filler particles has a larger number of filler particles. This enables more initial cracks to develop independently, which makes damaging of the specimen more difficult; whereas, in a random case, a larger number of filler particles makes filler particles aggregate, which leads to cracks connecting more easily and then a lower strength. As a result, the improvement of the strength brought by the larger number of filler particles is offset by this side effect in the case of the randomly-distributed filler particles.

In addition, it can be seen in Figure 8a,b that the strength of specimens with a weak filler-matrix interface (WI) is always much lower compared to that of specimens with a strong interface (SI). This indicates that the effect of the particle size on the strength is much smaller than the effect of the interface strength on the strength of the cement paste specimen.

### 3.3. Effect of Filler Shape

**Figure 9:**
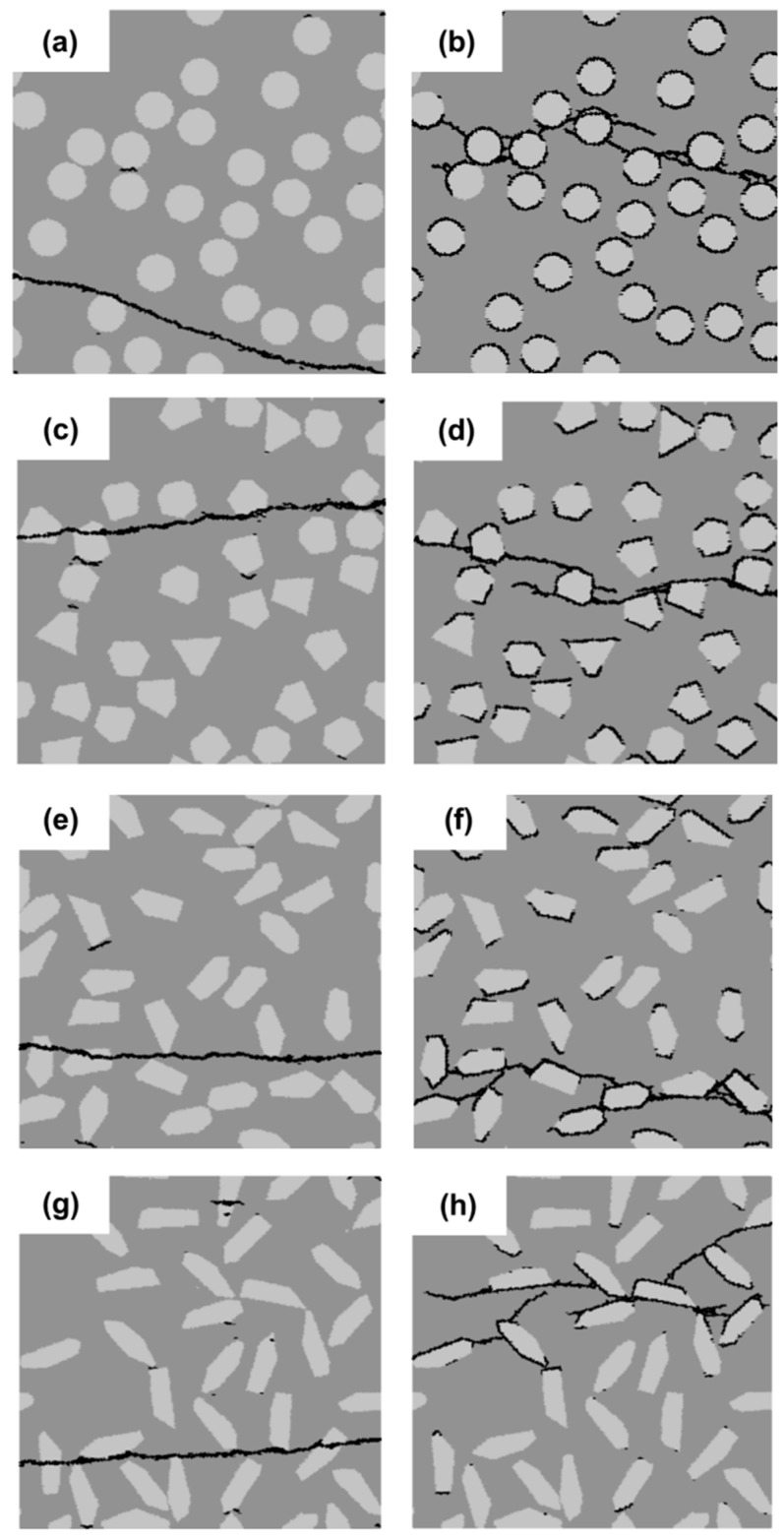
Micro-crack patterns of specimens with strong interface (left) and a weak interface (right) for four different shapes of filler particles (Shape 1 (**a**,**b**), Shape 2 (**c**,**d**), Shape 3 (**e**,**f**) and Shape 4 (**g**,**h**)).

In this section, the effect of the filler shape is investigated. Four different filler shapes are considered, as indicated in Figure 9. They are divided into two groups: on the left is the group with a strong interface (SI); on the right is that with a weak interface (WI). A circular particle shape (Shape 1), is used as a reference shape. The length-to-width ratio for arbitrary polygonal Shapes 2, 3 and 4 is about 1.0, 2.0 and 3.0, respectively. The filler area fraction is kept constant at 25%. The diameter of circular filler particles is 10 µm.

**Figure 10:**
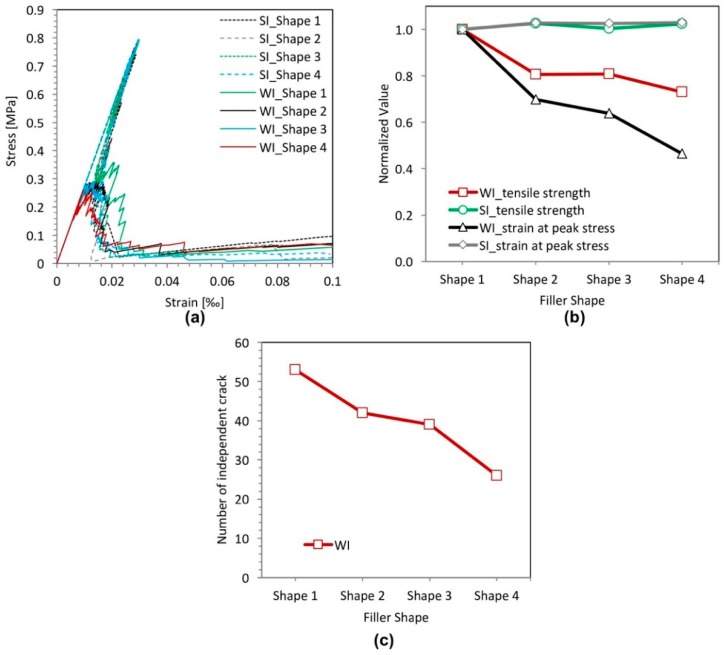
Simulated results of the filler shape effect analyses for specimens with a strong interface (SI) and a weak interface (WI). (**a**) Stress-strain relation; (**b**) variation of the strength and the strain at peak stress; (**c**) number of independent cracks.

Micro-crack patterns for the filler shape sensitivity analyses are shown in Figure 9. For the specimens with a strong filler-matrix interface (SI), one main crack and very few small cracks can be observed in Figure 9a,c,e,g, and the micro-crack patterns appear similar, except the direction of crack propagation. For the specimens with a weak filler-matrix interface (WI) (Figure 9b,d,f,h), the micro-crack patterns appear slightly different from each other. For the circular shape (Figure 9b), the branched cracks are uniformly distributed around the filler particles and tend to encircle the filler particles. For the arbitrary polygonal shapes (Figure 9d,f,h), the number of branched cracks around the filler particles decreases with the increasing length-to-width ratio of the particle. Moreover, with an increasing length-to-width ratio, it becomes increasingly difficult for cracks to propagate around and encircle the filler particles, preventing more initial-cracks from developing. Therefore, the number of independent cracks decreases with the increasing length-to-width ratio of the particle, as shown in Figure 10c.

Figure 10 plots the stress-strain relations (a), variation of the strength and the strain at peak stress (b) and the number of independent cracks (c) of the specimens with filler particles with different shapes. It is obvious that the filler shape has a small effect on the strength and the strain at peak stress of cement paste specimens with a strong filler-matrix interface (SI). On the contrary, for the cement paste specimens with the weak filler-matrix interface (WI), the filler shape has a significant influence on the strength of the specimens. The sharp edges of a polygonal particle shape are not favorable for initiation of cracks developing, which leads to the concentration of cracking, as shown in Figure 10c. Besides, the sharp edges of the polygonal particle shape also cause the stress concentration, which makes the specimen crack easily. Consequently, both the strength and the strain at peak stress of the specimens with circular filler particles are much higher than those with arbitrary polygonal filler particles. Furthermore, with an increasing length-to-width ratio of the arbitrary polygonal shapes, the strength and the strain at peak stress of the cement paste specimens with the weak filler-matrix interface (WI) appear to decrease.

Although the filler shape has a significant influence on the fracture behavior of the cement paste specimens with the weak filler-matrix interface (WI), the strength of these specimens is always much lower than that of specimens with the strong interface (SI), as shown in Figure 10a. This indicates that the filler-matrix interface strength is the dominant influencing factor on the strength of the cement paste specimens compared to the influence of filler particle shape.

### 3.4. Effect of Surface Roughness of Filler Particles

**Figure 11:**
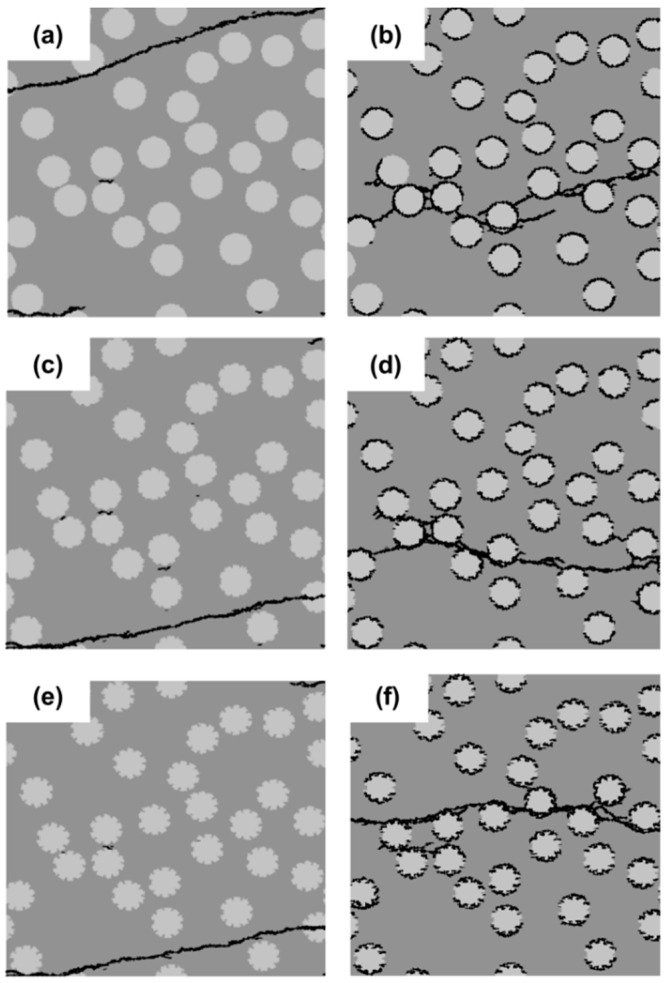
Micro-crack patterns of specimens with a strong interface (left) and a weak interface (right) for three different surface roughnesses of filler particles (Roughness 1 (**a**,**b**); Roughness 2 (**c**,**d**) and Roughness 3 (**e**,**f**)).

The surface roughness of the filler particles is one of the important factors determining the effect of mechanical interlocking between the filler particles and cement matrix [27]. In this section, the effect of the surface roughness of the filler particles on the fracture properties is analyzed. Three different surface roughnesses are considered, as indicated in Figure 11. They are divided into two groups: on the left is the group with a strong interface (SI); on the right is that with a weak interface (WI). The circular particle shape is used. The diameter of the filler particles is 10 µm. For particles with Roughness 1 (Figure 11a,b), the surface is smooth. For particles with Roughnesses 2 and 3, the surfaces are rough. The surface of particles with Roughness 3 (Figure 11e,f) is rougher than that of the particles with Roughness 2 (Figure 11c,d). The filler area fraction is kept constant at 25%.

**Figure 12:**
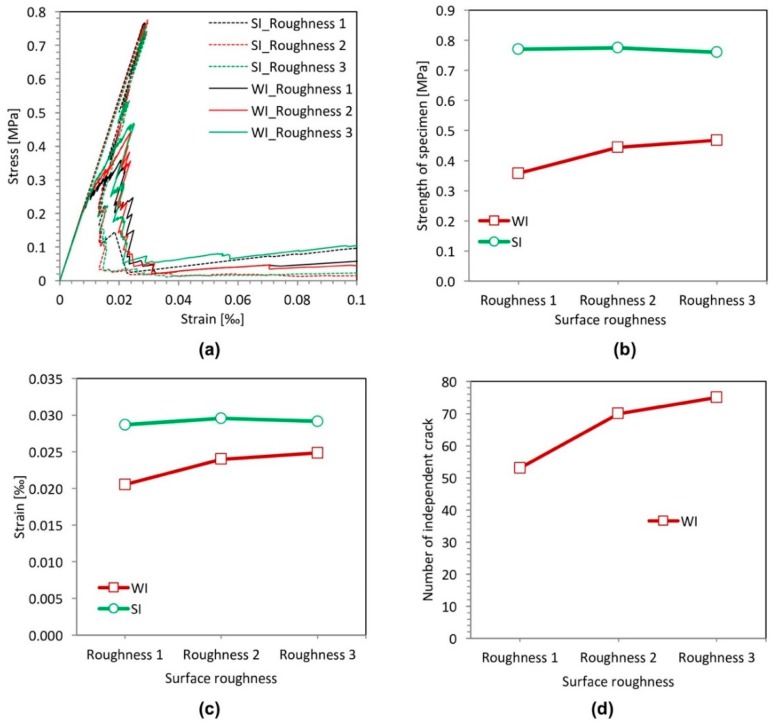
Simulated results of the filler surface roughness effect analyses for specimens with a strong interface (SI) and a weak interface (WI). (**a**) Stress-strain relation; (**b**) strength; (**c**) strain at peak stress; (**d**) number of independent cracks.

Figure 11 shows the micro-crack patterns of specimens with the filler particles at different surface roughnesses. For the specimens with a strong filler-matrix interface (SI) (Figure 11a,c,e), the micro-crack patterns appear similar, although the location of the crack for Roughness 1 is different. For the specimens with a weak filler-matrix interface (WI) (Figure 11b,d,f), the micro-crack patterns appear different. The number of independent cracks increases with increasing roughness, as shown in Figure 12d. This is due to the fact that the rougher surface makes initial cracks harder to connect.

Figure 12 shows the stress-strain relations, the strength, the strain at peak stress and the number of independent cracks for different particle surface roughnesses. The figure shows that in the specimens with a strong filler-matrix interface (SI), the surface roughness of the filler particles has little effect on the strength and the strain at the peak stress of cement paste specimens. For the cement paste specimens with a weak filler-matrix interface (WI), the surface roughness of the filler particles has a significant influence on the mechanical response of the cement paste specimen. Figure 12b,c shows that the strength and strain at the peak stress of specimens increase with increasing surface roughness. This is possibly because that the rougher surface makes initial cracks harder to connect and to cause final damage.

From the simulation, it is clear that in the specimens with a weak filler-matrix interface (WI), the surface roughness of the filler particles can improve the strength of specimens. However, even for the filler particles with the highest surface roughness, the specimens with a weak interface (WI) have a much lower strength than the specimens with a strong interface (SI), as shown in Figure 12b.

### 3.5. Effect of Filler Area Fraction

**Figure 13:**
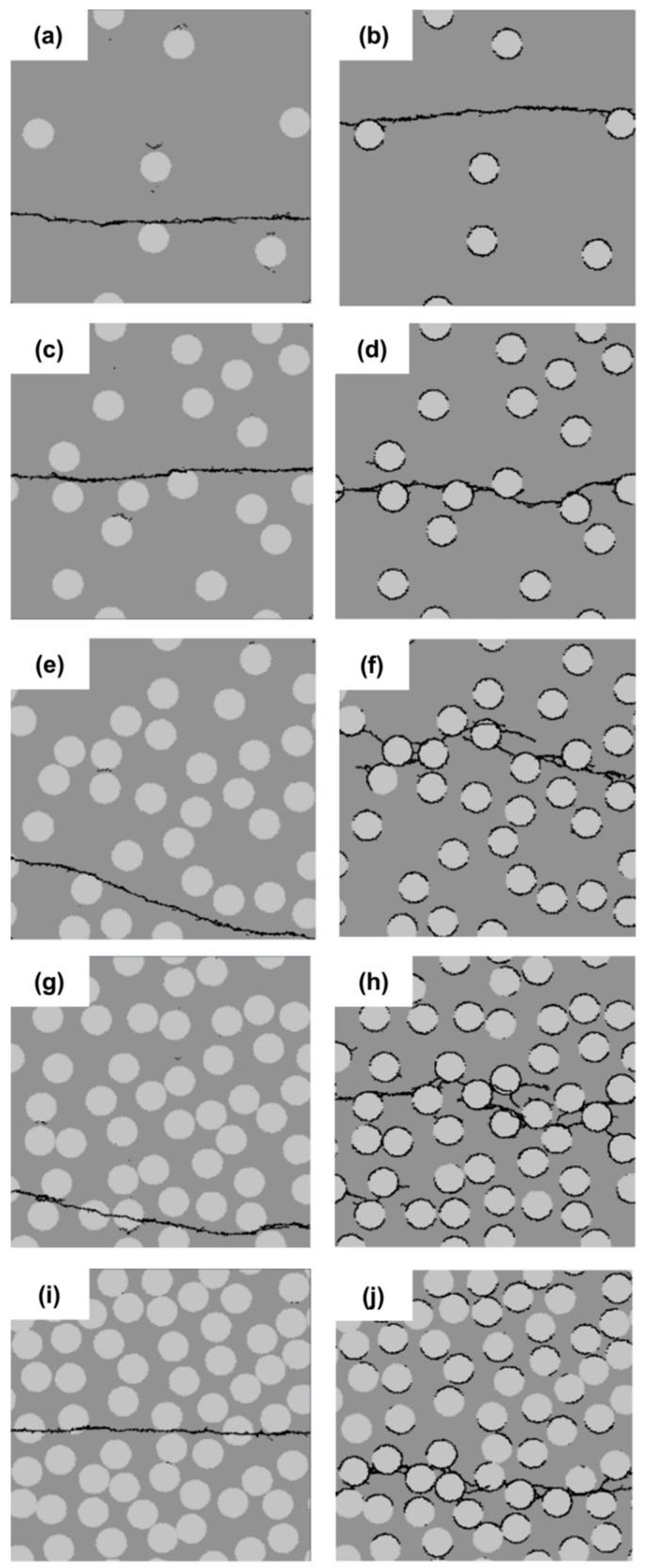
Micro-crack patterns of specimens with randomly-distributed filler particles and five different filler area fractions (5% (**a**,**b**); 15% (**c**,**d**); 25% (**e**,**f**); 35% (**g**,**h**) and 45% (**i**,**j**)).

**Figure 14:**
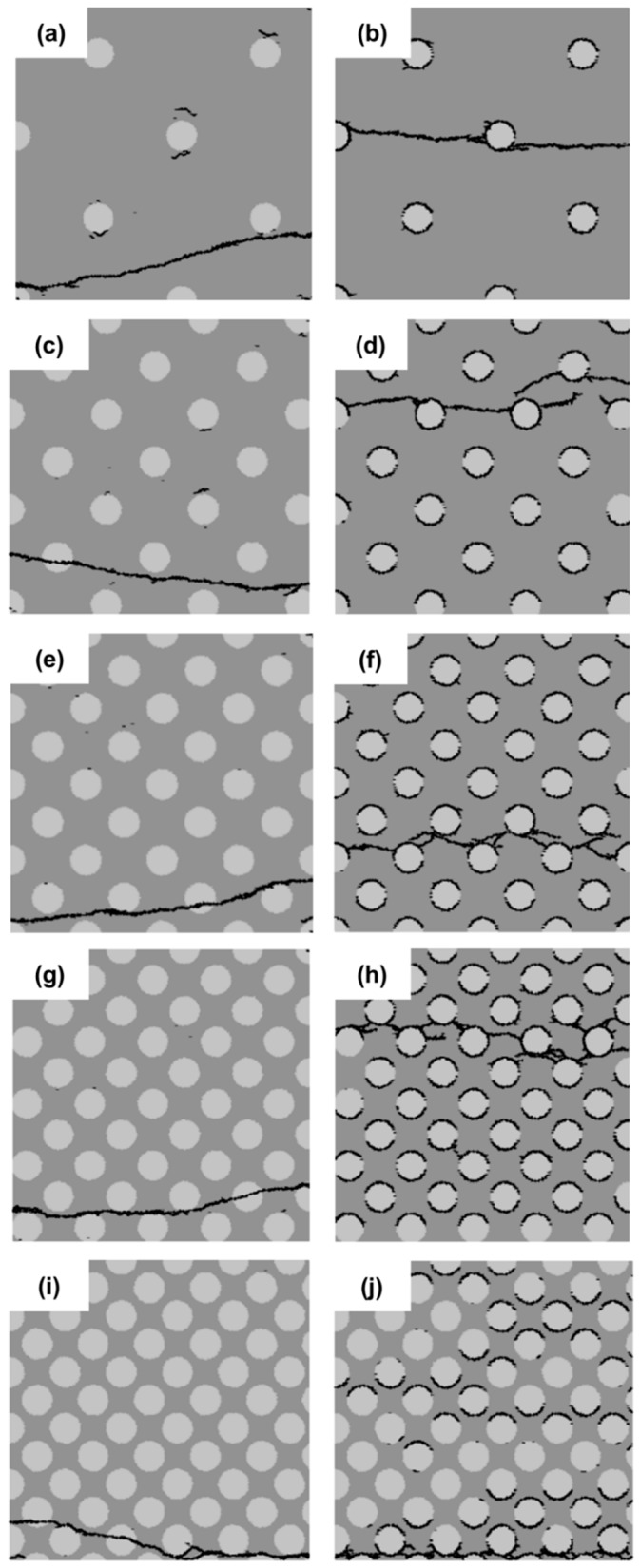
Micro-crack patterns of specimens with uniformly-distributed filler particles and five different filler area fractions (5% (**a**,**b**); 15% (**c**,**d**); 25% (**e**,**f**); 35% (**g**,**h**) and 45% (**i**,**j**)).

The effect of the filler content on the fracture behavior of cement paste specimens is examined in this section. Five different area fractions are considered: 5%, 15%, 25%, 35% and 45%. They are divided into two groups as indicated in Figure 13 and Figure 14: on the left is the group with a strong interface (SI); on the right is that with a weak interface (WI). The filler particles in Figure 13 are randomly distributed. As a reference, specimens are used with uniformly-distributed filler particles (Figure 14). The diameter of the filler particles is kept constant at 10 µm.

Micro-crack patterns of the specimens with different filler content are shown in Figure 13 and Figure 14. For the specimens with a strong interface (SI), the crack patterns in all specimens appear similar regardless of the filler content. For the specimens with a weak interface (WI), the crack patterns are different. For the specimen with 5% filler, the main crack is not branched, and the initial cracks develop well. For the specimen with a high filler area fraction (15%, 25%, 35% and 45%), the cracks are distorted and branched, and increasing initial cracks stop developing with an increasing filler area fraction.

**Figure 15:**
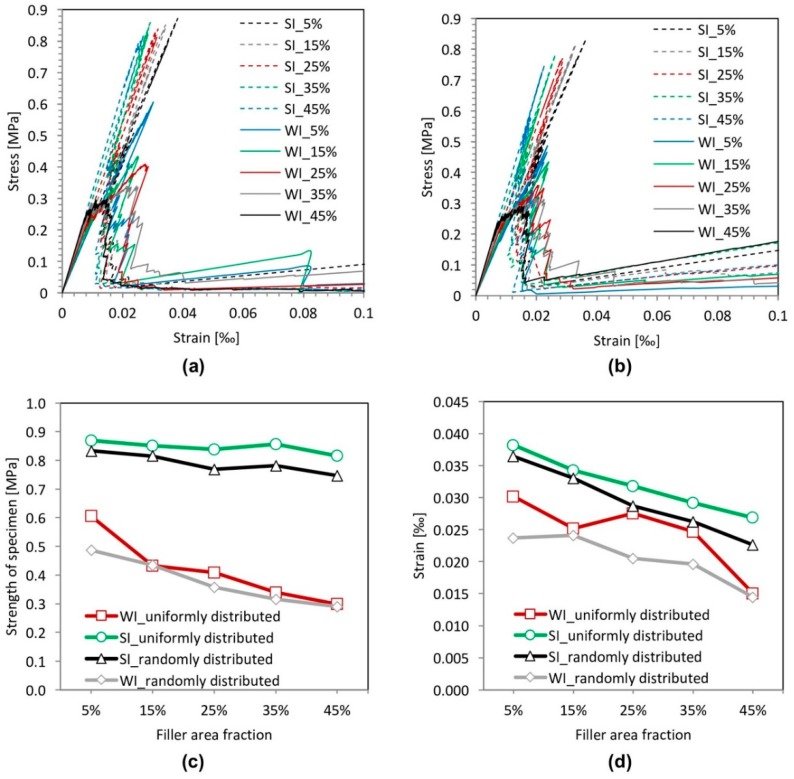
Simulated results of the filler area effect analyses for specimens with a strong interface (SI) and a weak interface (WI). (**a**) Stress-strain relation (uniformly distributed); (**b**) stress-strain relation (randomly distributed); (**c**) strength; (**d**) strain at peak stress.

The stress-strain relations, the strength and strain at peak stress for different filler content are shown in Figure 15. Figure 15c shows that the filler area has a smaller effect on the strength for specimens with a strong filler-matrix interface compared to the effect on the specimens with a weak filler-matrix interface. However, the strain at peak stress decreases with increasing filler area fraction (Figure 15d). This is because the Young’s modulus of cement paste specimens increases with increasing filler content. For the specimens with a weak interface (WI), the filler content has a significant influence on the strength. The strength decreases with increasing filler content. This is due to the increase of weak filler-matrix interfaces. This promotes propagation of cracks and, hence, reduces the strength of the cement paste specimens.

Although the filler content has a significant influence on the strength of specimens with a weak filler-matrix interface, the strength of specimens is always lower than that with a strong filler-matrix interface, as shown in Figure 15c. Even with a filler area fraction of 5%, the strength of the specimens with a weak interface (WI) is about 25% lower than that of the specimens with a filler area fraction of 45% with a strong interface (SI).

## 4. Conclusions

The influence of particle distribution, size, shape, surface roughness and area fraction of filler on the tensile strength and micro-crack pattern in blended cement paste specimens with strong and weak filler-matrix interfaces was systematically investigated by using the lattice model. Based on the numerical analyses, the following observations are made.

### 4.1. Strength of Cement Paste Specimens with a Weak Filler-Matrix Interface

- Randomly-distributed filler particles can decrease the strength of cement paste due to the aggregation of filler particles, which facilitates the connection of cracks and the final damage.
- In the investigated range of particle size of the filler (5 µm, 10 µm, 15 µm and 20 µm), the influence of the particle size on the strength of specimens is not noticeable.
- The sharp edges of the polygonal particle shape are not favorable for initiation of cracks, which leads to the concentration of cracking. Besides, the sharp edges of the polygonal particle shape also cause the stress concentration, which makes the specimen easily crack. Consequently, both the strength and the strain at peak stress of the specimens with circular filler particles are much higher than those with arbitrary polygonal filler particles.
- With increasing surface roughness, the strength of the specimens increases because the rougher surface makes initial cracks harder to connect and to cause final damage.
- With increasing filler content, the strength of the specimens decreases due to the increase of weak filler-matrix interfaces, which promotes propagation of cracks and hence reduces the strength of the cement paste specimens.

### 4.2. Strength of Cement Paste Specimens with a Strong Filler-Matrix Interface

- The particle distribution has an influence on the strength of specimens, but the influence is not remarkable. The strength of specimens with uniformly-distributed filler particles is only a little higher than that of specimens with randomly-distributed filler particles.
- In the case of the specimens with uniformly-distributed filler particles, the influence of the particle size on the strength of specimens is small. However, in the case of specimens with randomly-distributed filler particles, the strength decreases with increasing filler particle size.
- The particle shape, roughness and content of filler have little effect on the strength of specimens.
- The influence of particle distribution, size, shape, surface roughness and area fraction of filler on the strength of the specimens with a strong interface is much smaller than on the strength of the specimens with weak interface.

### 4.3. Micro-Crack Pattern

- With a weak interface, the crack patterns appear different, especially the number of independent cracks.
- With a strong interface, the crack patterns appear similar regardless of the particle distribution, size, shape, surface roughness and area fraction of filler, although the location of the main cracks may change.
- The micro-crack patterns of the cement paste specimens with a strong filler-matrix interface are very different from those of the specimens with a weak filler-matrix interface.

The numerical simulations indicate that the strength of the filler-matrix interface plays a more important role in the crack propagation and the strength of blended cement paste than the particle distribution, size, shape, surface roughness and area fraction of filler. This study indicates the direction for optimization of the performance of fillers in cement paste in view of microcracking and strength. To improve the performance of fillers in blended cement paste in term of strength, priority should be given to improving the bond strength between filler particles and hydration products, not to modifying the microstructural features of filler.

## Figures and Tables

**Table 1:** Mechanical properties of materials and interface.

Material Properties	Young′s Modulus E (GPa)	Tensile Strength ƒt (MPa)	*ν*
Filler	71	2.1	0.2
Matrix	22	1.4	0.2
Strong interface (SI)	17	1.4	0.2
Weak interface (WI)	17	0.4	0.2
